# EVALUATION OF AMINO ACIDS LEVELS OF HOSPITALIZED PATIENTS WITH DELIRIUM

**DOI:** 10.1093/geroni/igad104.1488

**Published:** 2023-12-21

**Authors:** Gohar Azhar, Shakshi Sharma, Regina Gibson, Amanda Pangle, Robert Wolfe, Jeanne Wei

**Affiliations:** University of Arkansas for Medical Sciences, Little Rock, Arkansas, United States; University of Arkansas for Medical Sciences, Little Rock, Arkansas, United States; University of Arkansas for Medical Sciences, Little Rock, Arkansas, United States; University of Arkansas for Medical Sciences, Little Rock, Arkansas, United States; University of Arkansas for Medical Sciences, Little Rock, Arkansas, United States; University of Arkansas for Medical Sciences, Little Rock, Arkansas, United States

## Abstract

Delirium is an acute and often fluctuating change in cognitive status characterized by disorientation, disorganized thought processes and changes in perceptual and psychomotor function. The common precipitating etiologies of delirium include any acute illnesses, infections, hospitalizations, fluid or electrolyte imbalance, or medications. Neurotransmitters are synthesized from amino acids. We hypothesized that regardless of the clinical reason for delirium, a sudden imbalance of plasma amino acids might alter the level of neurotransmitters and contribute to delirium. We measured amino acids in hospitalized patients 60 or older, both genders, with delirium secondary to any infection (n=13) for a period of 3 days and compared them with healthy control, non-delirious group (n=13). Delirium was evaluated twice daily using the Confusion Assessment Method (CAM) test. Plasma amino acids were analyzed using LC-MS. Our results showed that level of isoleucine was significantly higher in delirious patients on day 1 (64.26 %) as compared to healthy control (43.25%, p < 0.01). The level of tryptophan was slightly lower on day 1 as compared to control (31.13 % vs 36.80%, p=NS). However, the tryptophan/isoleucine ratio on day 1 was significantly lower in delirious compared to healthy controls (49.96% vs 85.54 %, p < 0.01). The tryptophan/isoleucine rose on day 2 of delirium but was still significantly lower compared to the healthy control (59.0% vs 85.54%, p < 0.01). Our results suggest that significant alterations in the levels and ratios of amino acids, in particular, tryptophan and isoleucine might disrupt the synthesis of neurotransmitters and hence contribute to delirium.

